# Prevalence of Systemic Lupus Erythematosus in Australia, 2010–2022: A Population‐Based Study Using Linked National Administrative Health Data

**DOI:** 10.1002/acr.80026

**Published:** 2026-04-07

**Authors:** Lucinda Roper, Rachel Black, Joanna Tieu, Winnie Chen, Matthew Parker, Catherine Hill, Natasha Nassar, Mandana Nikpour

**Affiliations:** ^1^ Sydney School of Public Health, Faculty of Medicine and Health The University of Sydney Sydney New South Wales Australia; ^2^ Leeder Centre for Health Policy, Economics and Data, Faculty of Medicine and Health The University of Sydney Sydney New South Wales Australia; ^3^ Royal Adelaide Hospital, Central Adelaide Local Health Network Adelaide South Australia Australia; ^4^ The Queen Elizabeth Hospital, Central Adelaide Local Health Network Adelaide South Australia Australia; ^5^ Faculty of Health and Medical Sciences The University of Adelaide Adelaide South Australia Australia; ^6^ Menzies School of Health Research, Charles Darwin University Darwin Northern Territory Australia; ^7^ Sydney Musculoskeletal Research Centre, Faculty of Medicine and Health The University of Sydney Sydney New South Wales Australia; ^8^ Department of Rheumatology Royal Prince Alfred Hospital Sydney New South Wales Australia; ^9^ Institute of Academic Medicine, Royal Prince Alfred Hospital Sydney New South Wales Australia; ^10^ Children's Hospital at Westmead Clinical School, Faculty of Medicine and Health The University of Sydney Sydney New South Wales Australia; ^11^ Charles Perkins Centre, The University of Sydney Sydney New South Wales Australia

## Abstract

**Objective:**

Systemic lupus erythematosus (SLE) is a heterogenous inflammatory condition with widely varying global prevalence estimates. The frequency of SLE in the general population of Australia has been reported to be notably lower than contemporary estimates in countries such as the United States or United Kingdom, at 19 to 39 per 100,000 as opposed to 65 to 97 per 100,000. This study aimed to develop a national SLE cohort using linked administrative data sets and to estimate prevalence using this approach.

**Methods:**

We developed an algorithm to identify SLE cases using the National Health Data Hub, a linked national administrative data asset, which includes data on medications dispensed and medical services subsidized under Australia's universal subsidized coverage schemes, hospital admissions, and deaths. These classification criteria were developed with an expert panel of rheumatologists. Individuals were classified as “certain,” “uncertain,” or “no SLE.” Cohort characteristics were described, and period prevalence (2010–2022) was calculated.

**Results:**

A cohort of 26,788 individuals with SLE were identified, which comprised 16,294 certain cases and 10,494 uncertain cases. The period prevalence of SLE in Australia from 2010 to 2022 was 77 to 127 per 100,000 (certain cases or overall cases). Among the certain cases, just over half had received care for SLE only in an outpatient setting.

**Conclusion:**

This is the first Australian SLE study using comprehensive linked administrative health data, and it identified higher prevalence than previously reported, more closely aligning with international estimates. This algorithm may serve as a foundation for future Australian and international studies seeking to identify SLE in administrative health data sets.

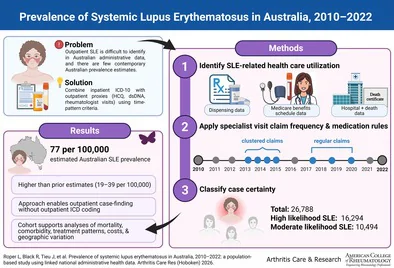

## INTRODUCTION

Systemic lupus erythematosus (SLE) is a heterogenous systemic autoimmune rheumatic disease (SARD), with female predominance and a wide variety of manifestations. The underlying etiology is thought to include both genetic and environmental factors.^1^ Tracking disease prevalence and regional variation can help uncover possible disease triggers and improve health equity by allowing service delivery to target areas with the most people affected. Given the increasing therapeutic options^2^ and evidence for a “treat to target” approach, the identification of a national cohort is valuable to allow the study and benchmarking of health outcomes.

**SIGNIFICANCE & INNOVATIONS**

- This article presents a novel electronic phenotyping algorithm for systemic lupus erythematosus (SLE) identification using administrative health data, applicable to settings where outpatient diagnostic codes are unavailable.
- Prevalence estimates identified using this cohort (77–127 per 100,000) were substantially higher than previously reported in Australia and comparable with international figures, suggesting previous under ascertainment.
- The resulting cohort, spanning hospital and nonhospital settings, enables research on the full spectrum of SLE severity and health care use.

Globally, there has been a broad range of prevalence estimates reported for SLE, from 5.05 to 241 per 100,000.^3^, ^4^ This likely reflects true regional variation related to environmental and genetic factors, as well as variation in data sources, definitions, and methodology. As outlined in our recent systematic review,^5^ there are limited Australian data on SLE prevalence, with only four studies of SLE in adults eligible for inclusion in a meta‐analysis. All of these were confined to northern Australia, with a specific research focus on identifying SLE among Aboriginal and Torres Strait Islander people. Furthermore, recruitment for most of these studies was from a single town or hospital, and many of these are two to three decades old. Meta‐analysis of the existing data estimated the prevalence of SLE in Australia at 19 to 39 per 100,000 in non‐Aboriginal Australians^6^, ^7^, ^8^, ^9^ and 54 to 166 per 100,000 in Aboriginal and Torres Strait Islander people. In contrast, the overall prevalence in the United States has been recently estimated at 65 to 81 per 100,000^10^ and the United Kingdom at 97 to 107 per 100,000.^11^, ^12^ Given its rarity, broad range of clinical manifestations, and spectrum of severity, as well as the diverse range of health care providers involved in diagnosis and management, high‐quality prevalence estimates for SLE are difficult to obtain. Most prevalence studies rely on a regionally confined census because random sampling approaches require prohibitively large sample sizes.

Use of administrative health data for case identification provides an alternative approach.^13^ Administrative health data typically encompass information collected during health care services by government agencies for administrative and billing purposes. Examples include hospital admission data and birth and death registries.^14^ These data can be linked to create a comprehensive record of an individual's health service use. Linked, population‐wide data sets offer a powerful tool for evaluating disease prevalence, patient characteristics, service use, and outcomes. Administrative data are widely used for disease identification, but SLE's clinical heterogeneity and varied management result in nonuniform health service use, which complicates case ascertainment. Furthermore, administrative health data sources capture different types of information, with some more related to health service contacts rather than diagnostic information. Over a decade ago, a systematic review outlined these challenges,^15^ and refining identification methods remains an ongoing area of research.^16^, ^17^, ^18^, ^19^, ^20^ No comprehensive Australian algorithms exist; most studies rely on a single diagnosis (usually based on an *International Classification of Diseases* [*ICD*] code), which has known limitations.^17^, ^21^

Alternative cohort or registry data sources may provide complementary information to help ascertain cases and study patterns of care. In Australia, the Optimising Patient Outcomes in Australian Rheumatology (OPAL) data set provides a source of real‐world data on patients.^22^ OPAL captures routine data from over 200 rheumatologists practicing in private and public settings.^22^ A 2023 OPAL study of 4,260 patients with SLE described medication use, rheumatologist visit frequency, and demographics.^22^ Although OPAL predominantly reflects private care, it remains a useful reference for understanding prescribing practices and frequency and timing of specialist visits. The aims of this study were to identify a national cohort of people with SLE using administrative health data, to describe cohort demographics and health care use, and to estimate the prevalence of SLE in Australia.

## MATERIALS AND METHODS

### Study population and design

This is a population‐based observational, data linkage study to identify and follow‐up with people diagnosed with or receiving health care for SLE in Australia from July 1, 2010, to June 30, 2022.

### Data sources

This study used a national linked administrative health data platform, the National Health Data Hub (NHDH), consisting of 11 data sets that are probabilistically linked at the person level.^23^ These data sets include information from national data collections and data collections from most Australian states and territories, including New South Wales (NSW), Victoria (VIC), Queensland (QLD), Tasmania (TAS), and South Australia (SA). All data are deidentified before researcher access, and the NHDH follows extensive data governance, privacy, and security protocols, and the consent requirement is waived.^23^ Five of the 11 NHDH linked data sets were used in this study and are summarized below and described in detail in Supplementary Material 1:

1. The Medicare Benefits Schedule (MBS) data set (Australia's universal subsidized health care system), including type, date, and cost of service but not including test results.
2. The pharmaceutical benefits scheme (PBS) data set, which captures data on medicines dispensed under the national subsidy scheme and includes most prescription medicines in routine clinical use in Australia. Unfortunately, belimumab has never been PBS‐listed, and anifrolumab was listed only after the study period, so the use of these biologics is not captured.
3. The National Death Index (death data), which includes facts and causes of death from statutory death registrations.
4. Admitted patient care data drawn from the National Hospital Morbidity Database (hospital data).
5. The National non‐Admitted Patient episode‐level (NAP) data set, including records for public hospital patients attending outpatient clinics.

There is variation in data availability by state: hospital and NAP data collections did not include data from Western Australia (WA) or the Northern Territory (NT), which comprise 11% of the Australian population. Private hospital data for NSW, TAS, and SA were absent, and the NAP data collection was absent for SA. The impact of this on the overall cohort size was estimated to be small (~4%) because of the layered nature of the algorithm (Supplementary Materials 2 and 3).

### Case identification

**Figure 1:**
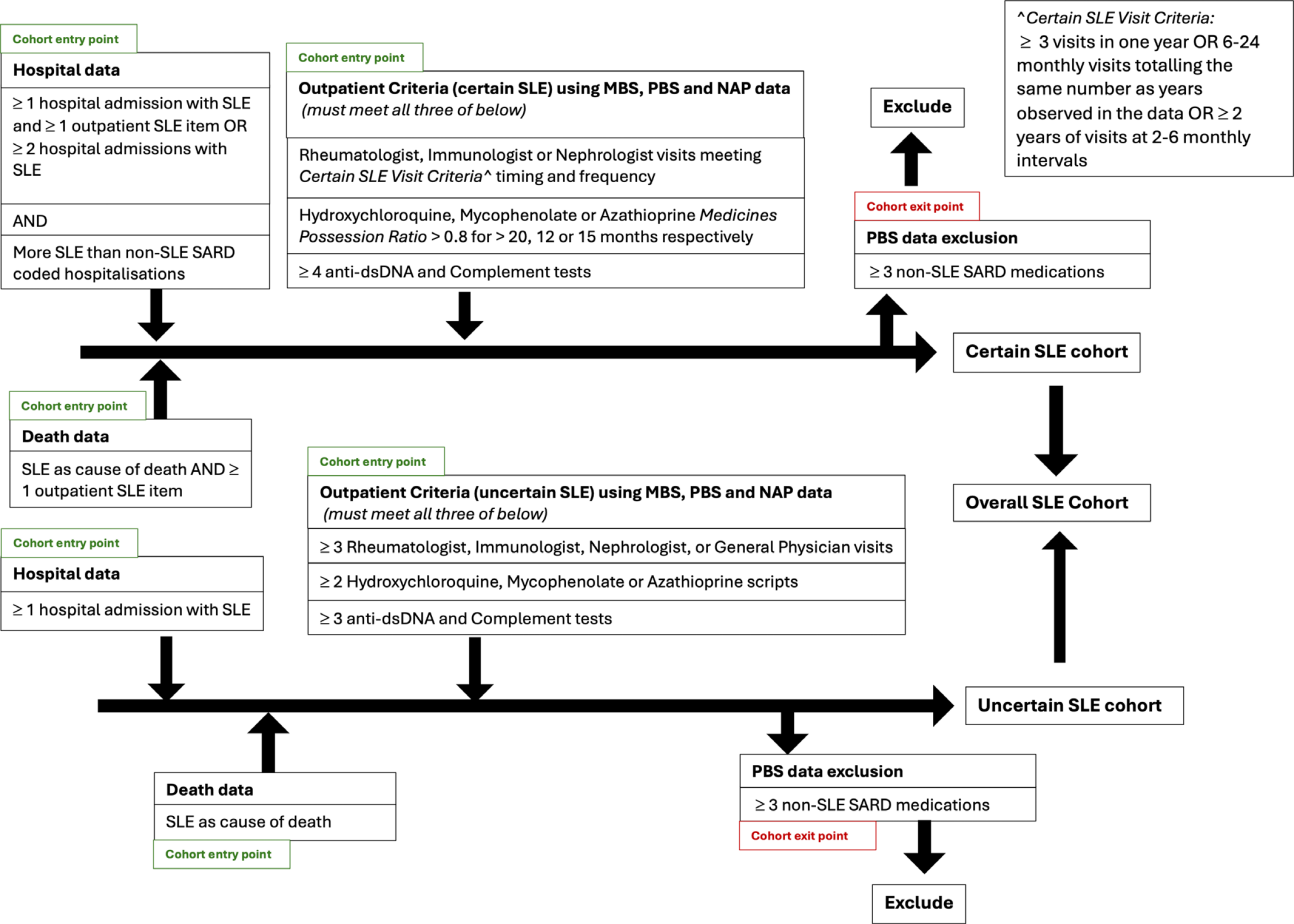
Overview of SLE classification algorithm. Supplementary Material 4 presents the figure in a detailed table format. anti‐dsDNA, anti–double‐stranded DNA antibodies; MBS, Medicare Benefits Schedule; NAP, non‐admitted patient episode level data; PBS, Pharmaceutical Benefits Scheme; SARD, systemic autoimmune rheumatic disease; SLE, systemic lupus erythematosus.

Individuals with SLE were identified using an algorithm detailing specific inclusion and exclusion criteria (Certain inclusion: two or more SLE hospital admissions and more SLE than non‐SLE SARD admissions; or one SLE hospital admission plus a non‐hospital SLE data point and more SLE than non‐SLE SARD admissions; SLE recorded on death data plus a non‐death SLE data point, or death before 2014; qualifying specialist visit patterns, medication exposure thresholds, and at least four dsDNA and complement tests. Uncertain inclusion: at least one SLE hospital admission and more SLE than non‐SLE SARD admissions; SLE recorded on death data; and at least three relevant specialist or general physician visits, at least two dispensings of HCQ, azathioprine or mycophenolate, and at least three dsDNA and complement tests. Exclusion: non‐SLE SARD listed as underlying cause of death where SLE was additional only; three or more dispensing of a non‐SLE approved DMARD (i.e. TNFi); failure to meet the relevant visit, dispensing, MPR, pathology, or corroboration thresholds.), consisting of a combination of SLE‐related hospitalizations, MBS claims, death records, and claims for dispensed medications (Figure 1 and detailed in Supplementary Material 4). The algorithm has two rule sets, classifying individuals as having “certain” or “uncertain” SLE, allowing the generation of upper and lower bound prevalence estimates. Sensitivity analyses tested the robustness of the algorithm by recalculating certain and uncertain SLE cohorts under alternative rule sets. Decisions that changed cohort size by more than 10% triggered a random review of 5% of affected records for adjudication of the rule based on clinician consultation (Supplementary Material 5). Table 1 illustrates the data items extracted from each data set, whereas Figure 1 illustrates how these were combined to create the SLE identification algorithm.

**Table 1:** SLE‐related diagnoses, medications, and health contacts extracted from each dataset*

Hospital data	Inclusion: Hospital admissions with SLE as a principal or additional diagnosis. *ICD‐10‐AM* codes M32.1, M32.8, M32.9. Exclusion: More admissions with a non‐SLE SARD diagnosis than an SLE diagnosis. Examples of SARD are rheumatoid arthritis, spondyloarthropathies, systemic sclerosis, vasculitis, and idiopathic inflammatory myopathies (full list in Supplementary Material 2).
Death data	SLE as the underlying or additional cause of death: *ICD‐10‐AM* codes M32.1, M32.8, M32.9 (details in Supplementary Material 6).
PBS data	Inclusion: Dispensing of hydroxychloroquine, azathioprine, or mycophenolate. Medication adherence criteria are outlined in Figure 1 and in detail in Supplementary Material 7. Exclusion: Chronic dispensing of medications that are not approved for use in SLE in Australia. Examples are biologic agents such as infliximab and adalimumab with restricted PBS indications to inflammatory diseases such as rheumatoid arthritis, ulcerative colitis, and psoriatic arthritis (full list in Supplementary Material 7).
MBS and NAP data	Outpatient visits to rheumatologist, nephrologist, immunologist, or general physician. Visit frequency requirements are outlined in Figure 1.
MBS data	Claims for testing anti‐dsDNA and C3 and C4. Testing frequency requirements are outlined in Figure 1 and Supplementary Material 8.

* anti‐dsDNA, anti–double‐stranded DNA antibodies; C3, complement component 3; C4, complement component 4; *ICD‐10‐AM*, *International Statistical Classification of Diseases and Related Health Problems*, *Tenth Revision*, *Australian Modification*; MBS, Medicare Benefits Schedule; NAP, National non‐admitted patient episode level data; PBS, Pharmaceutical Benefits Scheme; SARD, systemic autoimmune rheumatic disease; SLE, systemic lupus erythematosus.

### Algorithm justification

Hospital data criteria were informed by previously validated *ICD*‐based approaches for SLE identification,^15^, ^18^, ^19^, ^24^, ^25^ which demonstrate higher positive predictive value (PPV) with at least two SLE‐coded hospital admissions. We therefore required either multiple SLE‐coded episodes (hospital admissions) or a single SLE‐coded episode and a supporting outpatient SLE‐related claim or medication (eg, hydroxychloroquine) to meet our hospital criteria (Figure 1 and detailed in Supplementary Material 2). Death data criteria were based on *ICD* coded death certification (details in Supplementary Material 6). The Australian data sets that capture outpatient service use (MBS, NAP, and PBS) do not contain any diagnosis codes. We therefore used a pragmatic and innovative approach to identify individuals who had solely outpatient management of SLE (Figure 1), requiring them to meet criteria related to medication history, outpatient specialist visits, and pathology testing. These three criteria are outlined in Figure 1 and discussed below. These criteria were informed by real‐world data from the OPAL data set on how a subset of Australian patients with SLE were treated^22^ and how each criteria was reviewed, refined, and approved by the five rheumatologist authors, who have practiced across multiple Australian states in the public and private sectors.

1. Medication history (Supplementary Material 7): Most patients use hydroxychloroquine, mycophenolate, or azathioprine during their SLE treatment.^26^, ^27^ An individual was classified as “using” a medication if their medication possession ratio (MPR) was >0.8.^28^, ^29^ Calculation of the MPR is described in Supplementary Material 7. OPAL data informed certain SLE usage durations,^22^ with the certain SLE cohort requiring more than 20 months use of hydroxychloroquine, more than 15 months of azathioprine, or more than 12 months of mycophenolate. To avoid bias toward highly adherent individuals (real‐world data indicates that 51–90% of patients with SLE have an MPR >0.8),^30^, ^31^ the uncertain SLE inclusion rule was less stringent, requiring two dispensings of hydroxychloroquine, mycophenolate, or azathioprine only. Individuals with more than three dispensings of biologic or targeted synthetic medications that have PBS indications restricted to non‐SLE inflammatory conditions (ie, tumor necrosis factor [TNF] inhibitors or JAK inhibitors for rheumatoid arthritis or inflammatory bowel disease) were excluded. The threshold of three dispensings was selected based on clinical expert consensus that a short therapeutic trial with a non‐SLE medication may occur when there is initial diagnostic uncertainty. Glucocorticoid use was not required for enrollment because it is not specific for SLE.
2. Outpatient specialist visits (Supplementary Material 3): In Australia, patients with SLE are managed by rheumatologists, immunologists, nephrologists, and general physicians, particularly in rural areas. As per the expert clinician group consensus, stable patients are typically seen annually, with increased visits during flares; however, a subset have frequent flare‐related visits that are followed by long gaps in follow‐up. The certain SLE visit criteria identify both those with stable, regular visits and those with visit clusters between periods of minimal follow‐up. The uncertain SLE cohort only required three visits at any time during the study period.
3. Pathology testing (Supplementary Material 8): Anti–double‐stranded DNA antibodies (dsDNA) and complement level testing have been recommended to monitor SLE disease activity^32^ because fluctuations are associated with flares.^33^, ^34^, ^35^ Expert consultation suggested matching the number of dsDNA and complement tests to the minimum visit count per cohort, meaning that certain SLE required four tests, and uncertain SLE required three tests.

### Statistical analysis

Descriptive statistics for sociodemographic and health service use were reported for all SLE cases. Sociodemographic characteristics included sex, age at diagnosis, country of birth aggregated by region, geographic area of remoteness, and socioeconomic disadvantage quintile based on Australian Bureau of Statistics (ABS) Index of Relative Disadvantage.^36^ Measures of health care use comprising hospitalizations, medications dispensed, outpatient visits, and specialists seen were determined. Observation time was defined as the period from the first SLE‐related item (Table 1) to death or end of follow‐up (July 31, 2022). Details of these variables are in Supplementary Material 9.

Period prevalence (July 2010–July 2022) was calculated by dividing all identified SLE cases in each state or territory by the respective midpoint 2015 ABS population estimates, excluding WA and NT. To determine incidence rates, the annual number of SLE cases was calculated for each state and territory (excluding WA and NT) from 2015 to 2019, allowing for a four‐year lookback period to exclude prevalent cases and a two‐and‐a‐half‐year minimum follow‐up period. The incident date was defined as the first month an individual met the algorithm criteria, and annual incident cases were divided by the mid‐year ABS population estimates^37^ and reported per 100,000 in the population. Incidence rates are reported in Supplementary Material 10.

Individuals diagnosed before aged 15 years were excluded because the adult‐based algorithm may not apply due to different care patterns. Also excluded were individuals with data errors (eg, a data item appearing after death date or missing state of residence). For individuals who received care or who resided in multiple states, the first state was used for prevalence calculations.

All data management and analyses were done in R, version 4.4. Ethics approval for this study was obtained from the NSW Population and Health Services Research Ethics Committee (2024/ETH01955) after approval from NHDH data custodians (NG24‐0090).

## RESULTS

**Figure 2:**
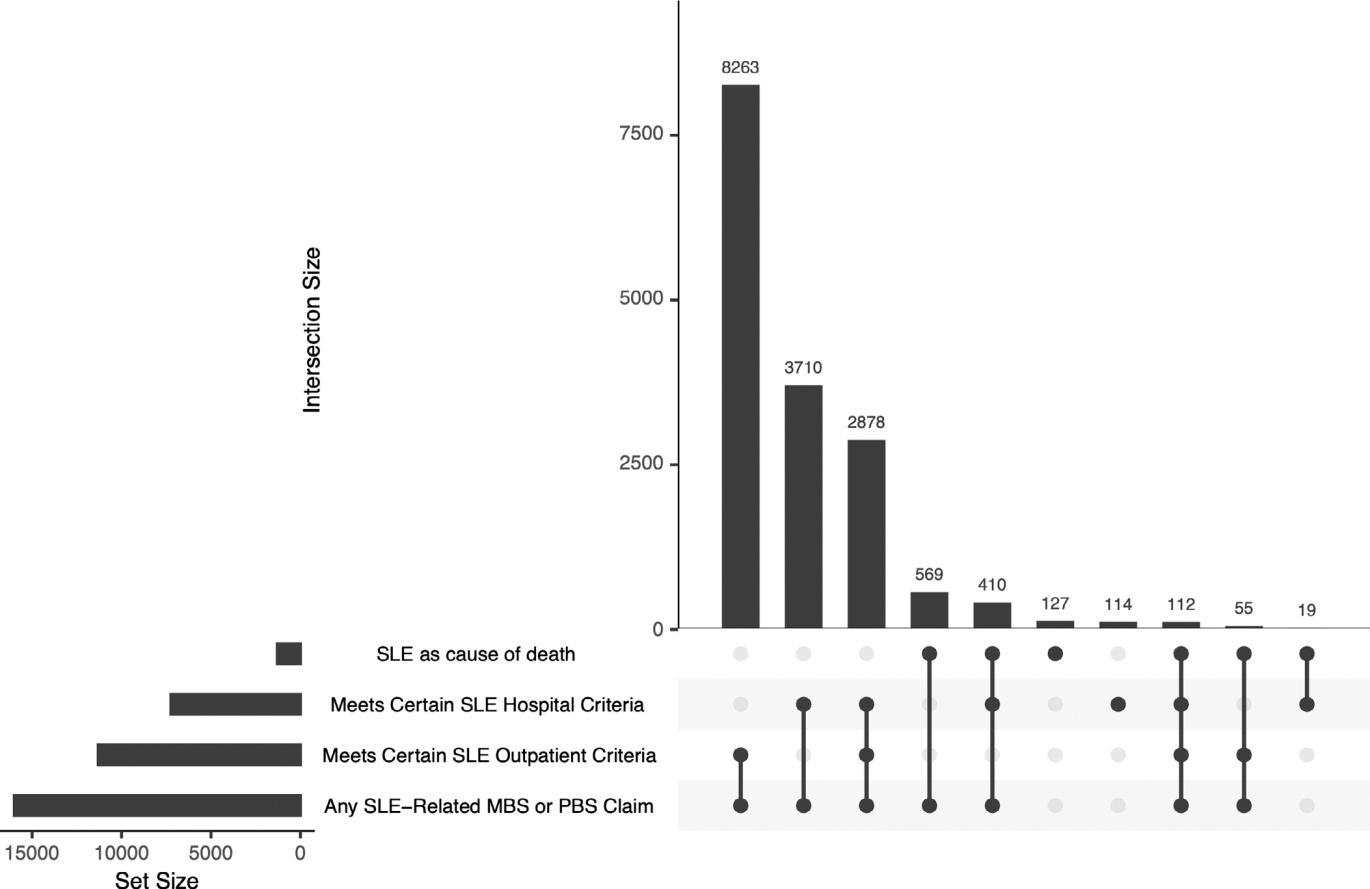
Horizontal bars represent the total people accessing the respective service; columns with circles represent each combination (darked circles), and vertical bars represent the number of people in the respective combination. For example, the first row represents the total number of people with records related to SLE in the death data set, whereas the first column represents the number of individuals fulfilling certain SLE criteria. Only the 10 most common intersections are shown. MBS, Medicare Benefits Schedule; PBS, Pharmaceutical Benefits Scheme; SLE, systemic lupus erythematosus.

A total of 26,788 SLE cases were identified, of which 16,294 were certain SLE. Of the certain cases, approximately half met outpatient criteria alone, and the remainder were identified by combinations of criteria as demonstrated in Figure 2. Table 2 presents sociodemographic characteristic of the certain, uncertain, and overall SLE cohort, who were predominantly female, with a younger age structure, higher proportion of migrants, and lower proportion of major city residence than the national average.^38^, ^39^

**Table 2:** Demographic characteristics of the identified SLE cohort*

Characteristics	Certain SLE, N = 16,294	Uncertain SLE, N = 10,494	Overall^a^ SLE, N = 26,788
Female, n (%)	14,099 (87)	8,991 (86)	23,090 (86)
Male, n (%)	2,158 (13)	810 (14)	2,968 (14)
Age at diagnosis, n (%), y^b^			
15–24	978 (6.0)	451 (4.3)	129 (5.3)
25–34	2,151 (13)	1,156 (11)	3,307 (12)
35–44	2,714 (17)	1,562 (15)	4,276 (16)
45–54	3,079 (19)	1922 (18)	5,001 (19)
55–64	3,143 (19)	2,189 (21)	5,332 (20)
65–74	2,602 (16)	1,855 (18)	4,457 (17)
75–84	1,326 (8.1)	1,073 (10)	2,399 (9.0)
85 and over	301 (1.8)	286 (2.7)	587 (2.2)
Region of birth, n (%)			
Australia/New Zealand	10,206 (63)	6,501 (62)	16,707 (62)
Europe	1,404 (8.6)	1,041 (9.9)	2,445 (9.1)
Asia	1973 (12)	884 (8.4)	2,857 (11)
Northern Africa and the Middle East	n.p.	n.p.	n.p.
Americas	n.p.	n.p.	n.p.
Sub‐Saharan Africa	203 (1.2)	112 (1.1)	315 (1.2)
Missing	1,949 (12)	1,606 (15)	3,555 (13)
Remoteness area, n (%)			
Major cities of Australia	10,393 (64)	6,555 (62)	16,948 (63)
Inner regional Australia	3,429 (21)	2,360 (22)	5,789 (22)
Outer regional Australia	2,055 (13)	1,322 (13)	3,377 (13)
Remote Australia	214 (1.3)	150 (1.4)	364 (1.4)
Very remote Australia	176 (1.1)	87 (0.8)	263 (1.0)
Missing	27 (0.2)	20 (0.2)	47 (0.2)
IRSD quintile, n (%)			
1 (most disadvantaged)	1,486 (20)	855 (19)	2,341 (20)
2	1,398 (19)	869 (19)	2,267 (19)
3	1,167 (16)	758 (17)	1925 (16)
4	1,724 (24)	1,086 (24)	2,810 (24)
5 (least disadvantaged)	1,510 (21)	1,014 (22)	2,524 (21)

* IRSD, Index of Relative Socioeconomic Disadvantage; n.p., not published; SLE, systemic lupus erythematosus.

^a^ Overall includes certain and uncertain SLE.

^b^ Age at meeting algorithm criteria.

Table 3 presents the health care use characteristics of the cohort. In the certain SLE cohort, median observation time from the first SLE‐related claim to censoring (death or July 31, 2022) was 12 years. There was a total of 2,356 deaths: 14.2 deaths per 1,000 person‐years. Just under half (46%) were ever hospitalized with SLE. The most dispensed medication was oral steroids, with 100% of the cohort receiving at least one script for oral steroids at some point during their median 11 years of observation. Similarly, most (88%) of the cohort received at least one script for hydroxychloroquine during the observation period. The proportion of the cohort taking key SLE medications is presented in Table 3. Because belimumab and anifrolumab were not PBS‐listed during the study period, data on their use are not available. Of the certain cohort, 72% had seen a rheumatologist, 31% had seen a nephrologist, and 24% had seen an immunologist. Other specialties frequently consulted included obstetricians, dermatologists, ophthalmologists, and general physicians.

**Table 3:** Health care use and management of individuals in the identified SLE cohort*

Health care utilisation and outcomes	Certain SLE, N = 16,294	Uncertain SLE, N = 10,494	Overall SLE, N = 26,788
Years observed total (post meeting algorithm)	12 (6)	10 (4)	11 (5)
Total deaths (death rate per 1,000 person‐years)	2,356 (14.2)	1,342 (13.9)	3,698 (14.1)
Hospitalizations			
Hospital admissions (any cause) per person, median (IQR)	5 (2–12)	4 (1–9)	5 (2–11)
Hospital admissions (SLE) per person, median (IQR)	0 (0–2)	0 (0–0)	0 (0‐1)
Ever hospitalized (any cause), %	88	85	87
Ever hospitalized with SLE, %	46	21	36
Receiving dialysis ever, n (%)	304 (2)	80 (1)	384 (1)
Receiving renal transplant, n (%)	344 (2)	65 (1)	409 (2)
Hospitalized for childbirth, n (%)	1,889 (12)	1,146 (11)	3,035 (11)
Medications – ever received, %			
Hydroxychloroquine	88	78	84
Azathioprine	22	13	19
Mycophenolate	25	12	20
Methotrexate	28	27	27
Oral prednisone	100	100	100
Cyclophosphamide	4	2	3
Leflunomide	5	6	5
Ciclosporin	2	1	2
Tacrolimus	4	1	3
Rituximab	1	1	1
IV methylprednisolone	5	4	4
Outpatient visits – ever seeing, %			
Rheumatologist	72	68	71
Nephrologist	31	18	26
Immunologist	24	22	23
General physician (internal medicine specialist)	53	51	52
Obstetrician/gynecologist	37	35	36
Dermatologist	37	37	37
Ophthalmologist	55	48	52
Ophthalmologist or optometrist for automated visual field testing	61	49	56
Annual GP (primary care doctor) visits, median (IQR)	10.2 (6.6–15.6)	10.1 (6.2–15.5)	10.2 (6.5–15.6)
Annual rheumatologist visits, median (IQR)	0.8 (0–1.8)	0.5 (0–1.3)	0.6 (0–1.6)

* GP, general practitioner; IQR, interquartile range; IV, intravenous; SLE, systemic lupus erythematosus.

Table 4 presents the 2010 to 2022 period prevalence of SLE. The period prevalence of SLE was 77 per 100,000 using the certain definition and 127 per 100,000 using overall cases.

**Table 4:** 2010 to 2022 period prevalence of SLE in Australia, using identified cohort*

State	Certain cases, n	Overall cases, n	Lower bound estimate (certain SLE) prevalence per 100,000	Upper bound estimate (overall SLE) prevalence per 100,000
ACT	454	764	115	193
NSW	6,614	10,669	87	140
QLD	2,984	4,950	62	104
SA	1,124	2,057	66	121
TAS	249	415	48	81
VIC	4,869	7,933	81	132
National, excluding WA/NT	16,294	26,788	77	127

* 2022 was a partial year. ACT, Australian Capital Territory; NSW, New South Wales; NT, Northern Territory; QLD, Queensland; SA, South Australia; SLE, systemic lupus erythematosus; TAS, Tasmania; VIC, Victoria; WA, Western Australia.

## DISCUSSION

This study describes the development of a detailed algorithm to identify Australians with SLE using linked administrative health data sets and incorporating SLE‐related hospital admissions, MBS claims, death records, and claims for dispensed medications. To the authors’ knowledge, it is the first algorithm to identify SLE cases using outpatient data in a jurisdiction without diagnostic coding of outpatient events and may serve as a template for other Australian or international researchers in jurisdictions without outpatient diagnostic codes. Using this algorithm to identify SLE, we estimate the prevalence of SLE in Australia to be 77 to 127 per 100,000, which is higher than previously reported^6^, ^7^, ^8^, ^9^ but similar to the contemporary findings in the United States of 81 per 100,000^10^ and the United Kingdom of 97 to 107 per 100,0000.^11^

The SLE cohort was predominantly female, which is consistent with established SLE cohorts.^22^ Compared with the Australian population, a lower proportion of the certain SLE cohort were born in Australia and New Zealand (63% vs 72%^39^), partly because of higher representation from Asian countries (12% vs 10.6%).^39^ The cohort also had greater regional representation (63% vs 72% in major cities^38^). The distribution of socioeconomic disadvantage was broadly consistent with national figures.

The SLE cohort demonstrated higher health care use than the general Australian population, despite a younger age profile. Median general physician (GP) visits were 10.2 per year compared with a national average of 6.3.^40^ Over the study period, 88% had at least one hospital admission for any indication (although less than half had a hospital admission for SLE care). Although not directly comparable with national annual rates because of the study observation period, this suggests higher hospital use than the general population—for which only 12.7% of Australians aged 15 and over were admitted to the hospital in 2023 and 2024.^41^

The SLE cohort demonstrated similar medication usage to confirmed Australian and international SLE cohorts,^22^ with similar hydroxychloroquine (88% vs 85% OPAL^22^) and methotrexate use (28% vs 29% OPAL^22^). Only half of our cohort had an MBS claim for visual field testing (a component of hydroxychloroquine screening that is advised in current Australian and New Zealand guidelines^42^). Oral steroid use was high in our cohort compared with the OPAL cohort, with 100% filling at least one prescription over the median 11‐year observation period versus 65.8% of the OPAL^22^ cohort reporting glucocorticoid use over 2.3 years. This likely reflects both differing definitions of steroid use and the longer observation period for our cohort. In OPAL, medications are recorded into a bespoke electronic medical record by a rheumatologist. In this context, steroids may be likely to represent maintenance glucocorticoids or current usage. Even if never prescribed maintenance glucocorticoids, most patients with SLE will require steroids for flares at some point—ie, 82% of the Asia Pacific Lupus Collaboration cohort used oral prednisone at least once over just two years.^43^ Short courses prescribed by a GP for a minor flare between rheumatologist visits may not be recorded in OPAL but would appear in PBS data, as would prescriptions filled for contingency use (ie, travel) and potentially not taken. Our longer timeframe also increases the likelihood of capturing a dispensing.

Although analysis of birth outcomes was outside the scope of the present study, at least 12% of the cohort had a hospital admission for birth. Finally, the mortality rate in SLE was 14.2 per 1,000 person‐years, compared with 18.6 per 1,000 person‐years in a recent Italian population‐based study^44^ or 15.84 per 1,000 person‐years in the United Kingdom.^45^

Although there are currently no validated algorithms for the identification of SLE from Australian administrative data, there has been substantial work in this space internationally, which informed our method. First, hospital data alone captures only 40% to 79% of SLE cases identified using clinician medical record review as the gold standard.^18^, ^25^ This persists even over long periods of observation (remaining at 79.2% after 40 years in a Swedish study^24^). This likely reflects the exclusion of younger and milder cases never requiring hospitalization and was further demonstrated by a Canadian study demonstrating that 32% of SLE cases identified in Quebec were present in outpatient claims data but absent from hospital records.^25^ These findings support our inclusion of both hospital and outpatient data as a methodologic strength.

Second, the PPV of a single SLE‐coded hospital or outpatient encounter is low but increases with repeat episodes. In Canada, the PPV of a single episode of SLE‐coded hospital care was 60%, rising to 87.5% with two or more hospitalizations.^25^ Similarly, in Norway, the PPV of a single SLE‐coded inpatient or outpatient encounter was 47%, increasing to 72% for three or more SLE‐coded episodes.^17^ Therefore, our hospital criteria require either multiple SLE‐coded episodes or an additional supporting claim from a different data source (eg, hydroxychloroquine). Third, clinical heterogeneity, diagnostic complexity, overlapping medication use, and shared specialist care contribute to the misclassification of SARDs in administrative data (for instance, the misclassification of rheumatoid arthritis as SLE). In Norway, half of the false‐positive SLE‐coded encounters were ultimately diagnosed as another SARD.^17^ Similarly, in Estonia, most false‐positive SLE‐coded care episodes were revised to an alternative SARD.^19^ These findings highlight the need for exclusion criteria to minimize SARD misclassification. We applied medication‐based exclusions (excluding individuals with SARD‐specific but non‐SLE therapies, such as TNF inhibitors) and hospital exclusion criteria (more admissions coded as non‐SLE SARD than SLE) to reduce this risk. Although these exclusions improve the specificity of the algorithm, SLE has been reported to overlap with many SARDs,^46^ and these cases may have been excluded if their clinical phenotype or management more closely aligned with the non‐SLE diagnosis (ie, had more hospital admissions coded as the non‐SLE diagnosis or received TNF inhibitor).

A key strength of this study is that it uses population‐wide, linked administrative data sets to identify individuals with likely SLE that are informed by existing Australian data as well as international SLE case‐finding algorithms, as described previously. Second, Australia's universal health care system enables near‐complete capture of real‐world health service and medication use via the PBS and MBS data sets, which provides a comprehensive view of this cohort's health care use. Finally, most studies using administrative health data to identify health conditions make assumptions regarding the health care use of individuals with that illness, and the quality of the case identification is dependent on the quality of those assumptions. Our algorithm was informed by both international SLE identification algorithms,^17–19,26^ contemporary Australian data on real‐world SLE care,^22^ and the expert guidance of five rheumatologists with broad experience across public and private sectors in three states.

Although we would ideally have validated our approach with an additional external data set or through review of medical records of cases and noncases, this was not possible. At the time of analysis, the NHDH data governance policies precluded reidentification of records because of privacy principles. In addition, linkage to external SLE registry data would be inadequate because of a lack of noncase records. Nevertheless, we are confident in the value of our approach despite being unable to calculate the sensitivity or specificity of the algorithm. To test the internal validity of our algorithm, we conducted nine separate sensitivity analyses, each varying a single decision rule (Supplementary Material 5). The cohort size and characteristics remained relatively consistent, suggesting that our findings are not unduly influenced by specific parameter choices. However, future validation of this algorithm by reviewing the medical records of a subset of the cohort would be valuable if this becomes possible. We also cannot assume our algorithm has identical performance to those developed in different settings.^15^, ^18^, ^19^, ^24^, ^25^ Structural differences in health systems and data collected (eg, use of insurance claims data^15^ or outpatient diagnostic codes,^18^, ^19^ which are not collected in Australia) limit direct comparison with international administrative data SLE identification approaches. Furthermore, diagnostic coding systems (ie, *ICD*/International Classification of Primary Care/Systematized Nomenclature of Medicine) and their accuracy varies, influenced by factors such as clinical coders’ expertise (which may differ by national training standards^47^), as well as hospital‐specific quality of medical record keeping. Despite these differences in data sources and settings, we were able to calculate similar estimates for SLE to other settings.

Furthermore, although the NHDH has broad coverage, it was not universal. Private hospital and NAP data were incomplete; however, sensitivity analyses suggest minimal impact on overall cohort size because of the layered design of the algorithm. Multiple data sets were missing for the NT and WA, leading to their exclusion. SLE prevalence has been reported to be higher in Aboriginal and Torres Strait Islander people than non‐Indigenous Australians,^5^, ^48^ and WA and the NT have higher proportions of Aboriginal and Torres Strait Islander people than Southern Australia (31% of NT and 4% of WA in 2021 census vs 3.5% of Australia excluding these states^37^). Given these states are small (NT contains <1% of the Australian population and WA approximately 11%), their overall effect on the national estimate is likely minimal, and in the direction of an underestimation. Given their unique demographics, our prevalence figures cannot be presumed to apply to these states, particularly not to the NT.

Future work using this cohort may include and assessment of the geographic distribution of SLE, hospitalizations, and causes of death, and—with appropriate consultation—estimation of disease burden in Aboriginal and Torres Strait Islander people (work outside the scope of our current ethics approvals). Because the NHDH is regularly updated, it will soon be possible to examine trends in use of newer therapies, such as anifrolumab, which were not PBS‐listed during the study period.

This study presents the first Australian algorithm to identify SLE using both inpatient and outpatient administrative data. In doing so, it demonstrates that the national prevalence of SLE is higher than previously reported and aligns more closely with international estimates. The cohort identified displays high face validity, with demographics, medication usage, and health care use patterns comparable with those observed in existing Australian data. By detailing our methodology, including its strengths and limitations, we aim to support transparency and reproducibility. This algorithm may serve as a foundation for future Australian and international studies seeking to identify SLE in administrative health data sets.

## AUTHOR CONTRIBUTIONS

All authors contributed to at least one of the following manuscript preparation roles: conceptualization AND/OR methodology, software, investigation, formal analysis, data curation, visualization, and validation AND drafting or reviewing/editing the final draft. As corresponding author, Dr Roper confirms that all authors have provided the final approval of the version to be published and takes responsibility for the affirmations regarding article submission (eg, not under consideration by another journal), the integrity of the data presented, and the statements regarding compliance with institutional review board/Declaration of Helsinki requirements.

## Supporting information


**Disclosure Form**:

**Data S1** Supporting Information
