# Supplementary material for: Prevalence of Systemic Lupus Erythematosus in Australia, 2010–2022: A Population‐Based Study Using Linked National Administrative Health Data
Source: Arthritis Care Res (Hoboken). 2026 Apr 7;78(10):1438–48. doi: 10.1002/acr.80026 (PMC13613255; doi:10.1002/acr.80026)
Supplement: Supplementary file 2 [file ACR-78-1438-s001.docx]

**Prevalence of Systemic Lupus Erythematosus in Australia, 2010–2022: A Population-Based Study Using Linked National Administrative Health Data: Supporting Information**

[**Supplementary Material S1:** National Health Data Hub Datasets	1](#_Toc220857028)

[**Supplementary Material S2:** Hospital criteria detail	4](#_Toc220857029)

[**Supplementary Material S3:** Visitation criteria detail	7](#_Toc220857030)

[**Supplementary Material S4:** Overall algorithm	11](#_Toc220857031)

[**Supplementary Material S5:** Selected sensitivity analyses of entire algorithm	13](#_Toc220857032)

[**Supplementary Material S6:** Death criteria detail	15](#_Toc220857033)

[**Supplementary Material S7:** Medication criteria detail	16](#_Toc220857034)

[**Supplementary Material S8:** Pathology criteria detail	25](#_Toc220857035)

[**Supplementary Material S9:** Demographic and disease characteristics	27](#_Toc220857036)

[**Supplementary Material S10:** SLE annual incidence rates	30](#_Toc220857037)

### **Supplementary Material S1:** National Health Data Hub Datasets

The following five NHDH linked datasets were used in this study:

(1) The Medicare Benefits Schedule (MBS) data for all of Australia. The MBS is Australia’s universal government subsidised healthcare system. The MBS dataset includes information on general practitioner, specialist and allied health consultations, medical procedures, and imaging, and pathology services. Location, date and cost of service is included, but results (i.e. pathology results) are not.
(2) The Pharmaceutical Benefits Scheme (PBS) data for all of Australia. The PBS dataset captures records of prescription medicines dispensed under the national subsidy scheme. This includes most prescription medicines in routine clinical use across the country. It details the medicine name, strength, quantity dispensed, dispensing date, and prescriber specialty.
(3) The National Death Index (NDI) for all of Australia, comprising records of all registered deaths in Australia, including the date of death and causes coded according to the ICD10-AM.
(4) Admitted patient care data drawn from the National Hospital Morbidity Database (hospital data), for public hospitals in New South Wales, Victoria, Queensland, Tasmania, the Australian Capital Territory and South Australia. Data from private hospitals in Queensland, the Australian Capital Territory and Victoria are also included. The NHMD is comprised of episode level records of each hospital admission. Each admission is assigned a principal ICD-10AM diagnosis code reflecting the main reason for care, with additional 50 diagnoses recorded for other conditions that influence the episode of care. Other information, such as bed days, hospital type, ICU time, funding status and admission dates are also included.
(5) The National Non-Admitted Patient episode-level Databases (NAP data) for New South Wales, Victoria, Queensland, Tasmania and the Australian Capital Territory. NAP data includes episode-level records for non-admitted patients, registered for care in public hospitals (for example attending hospital outpatient clinics). This includes information on hospital type, clinic type, date and funding status of service.

### **Supplementary Material S2:** Hospital criteria detail

The ICD10-AM codes used to select SLE and non-SLE SARD admissions re below:

| **Hospital and death data: ICD10-AM** | | |
| --- | --- | --- |
| SLE diagnosis codes | M32.1 | Systemic lupus erythematosus with organ or system involvement |
|  | M32.8 | Other forms of systemic lupus erythematosus |
|  | M32.9 | Systemic lupus erythematosus, unspecified |
|  |  |  |
| Non-SLE SARD diagnosis codes | M05.00-M05.99 | Seropositive rheumatoid arthritis |
|  | M06.00-M06.39,  M06.80-M06.99 | Other rheumatoid arthritis (excluding M06.40-M06.49 inflammatory polyarthropathy) |
|  | M30.0-M30.8 | Polyarteritis nodosa and related conditions |
|  | M31.0-M31.9 | Other necrotising vasculopathies |
|  | M08.00 – M08.99 | Juvenile arthritis |
|  | M09.00 – M09.29 | Juvenile arthritis in diseases classified elsewhere (excluding M09.9 Juvenile arthritis in other diseases classified elsewhere) |
|  | M33.0-M33.2, M33.9 | Dermatopolymyositis (juvenile dermatomyositis, other dermatomyositis, polymyositis, dermatopolymyositis, unspecified) |
|  | M34.0-M34.9 | Systemic sclerosis |
|  | M35.2 | Behcet’s disease |
|  | M35.3 | Polymyalgia rheumatica |
|  | M35.4 | Diffuse (eosinophilic) fasciitis |
|  | M35.5 | Multifocal fibrosclerosis |

The following definitions of hospital criteria were used in the overall sensitivity analysis.

| Hosp A | >=1 admission SLE + more SLE than SARD admissions + at least one other non-hospital data point.  OR  >=2 SLE admission + more SLE than SARD admissions |
| --- | --- |
| Hosp B | Any SLE hospital admission + more SLE than SARD admissions |
| Hosp C | >=1 admission SLE principal diagnosis or >= 2 admissions SLE any diagnosis code + more SLE than SARD admissions + at least one other non-hospital data point.  OR  >=2 SLE principal admission + more SLE than SARD admissions |

There was a total of 1,605 individuals identified who had a hospital admission with SLE and with a non-SLE SARD diagnosis code during the study period. To develop a rule for classifying these individuals as having SLE, we tested several thresholds:

- Excluding anyone with *any* non-SLE SARD admission;
- Requiring ≥75% of all musculoskeletal admissions to be coded as SLE;
- Requiring ≥50% of all musculoskeletal admissions to be coded as SLE.

For each rule, one author with clinical background reviewed a 5% sample of episode level linked data. The 50% threshold retained a group that, on clinical review of their hospital records (reviewing the principal and all additional diagnosis codes, the procedure codes, admission duration, overall frequency and type of admissions), appeared consistent with SLE. This group also had high rates of supporting outpatient indicators, including 88% usage of SLE-related immunosuppressive medications (AZA, MMF, or HCQ) and 93% undergoing serial anti-dsDNA testing.

**Missing private hospital data**

Private hospital data was only available for VIC, QLD and the ACT. The proportion of cases identified exclusively through private hospital data was calculated for these. Overall, whilst 10% of hospital admissions for SLE occurred in the private sector, less than 2% of the total cohort were identified exclusively using private hospital data, reflecting the built in redundancy in the algorithm. This represented 3% (certain) and 2.8% (uncertain) of the cohort of QLD, VIC and the ACT. Therefore, we have not applied an adjustment for the other states for assessing their prevalence but acknowledge that it may be 2-3% higher than reported.

### **Supplementary Material S3:** Visitation criteria detail

The following codes were used to identify specialist visits. All Medicare claims for a Medicare Australia Broad Type Of Service ‘Specialist Attendances’ (0200) (<https://meteor.aihw.gov.au/content/604330>) for the specialties with the Servicing Provider Registered Specialty codes listed below, were used to identify a specialist visit. Furthermore, all NAP data service episodes to one of the an immunology, rheumatology or nephrology clinic, with codes defined below, were used to identify a specialist visit.

| **MBS: servicing provider registered specialty** | |
| --- | --- |
| 003, 027, 083 | Immunology & Allergy,  Immunology,  C/Physician – Immunology and Allergy |
| 013, 093 | Rheumatology,  C/Physician - Rheumatology |
| 008, 088 | Nephrology,  C/Physician - Nephrology |
| 081, 082 | Consultant Physician – Internal Medicine,  Consultant Physician – General Medicine |
| **NAP data: OUTPATIENT_CLINIC_TYPE_TIER2** | |
| 20.30 | Outpatient clinic type: rheumatology |
| 20.35 | Outpatient clinic type: nephrology |
| 20.41 | Outpatient clinic type: immunology |

Outpatient visits to rheumatology, immunology, or nephrology clinics are usually billed to Medicare, depending on factors such as referral pathway, hospital funding arrangements, and whether a registrar or consultant conducted the review. It is important to consider this to avoid double counting specialist visits in the MBS and NAP data. The standard approach to this known issue is to define a single visit based on date of service. i.e. if an individual had an MBS code for seeing an immunologist and a NAP code for attending an immunology clinic, on the same date, this was counted as a single visit. The NHDH does not provide exact dates routinely, only months and years. Therefore, for a given month, the overall visit count was defined as: the larger of the total MBS codes or total NAP codes for a given specialty. This approach assumes that NAP and MBS codes are always paired, with additional NAP codes representing non-MBS billed admissions, and additional MBS codes representing non-NAP (i.e. private practice) physician attendances.

**Missing NAP data**

Non-admitted patient (NAP) data became available for ACT and TAS from 2013–14, for VIC and QLD from 2010–11 to 2016–17, and for NSW from 2017–18. The impact of this on the cohort was minimal, as the proportion of overall rheumatologist, nephrologist or immunologist specialist visits which were *only* identifiable through the NAP data (i.e. non-MBS billed clinic visits) was small. Furthermore, once our visit rules were applied, there were no individuals who met criteria from combined MBS and NAP data who would have been missed if NAP data was non-existent. This reflects the fact that our visit criteria have a high degree of redundancy built in.

The following visitation criteria were used in the sensitivity analyses displayed in Supplementary Material S6.

| Visit A | Rheumatologist, immunologist or nephrologist in MBS or NAP:  3 or more visits in 12-month period  OR  Total regular visits (defined as 6-24 monthly >= total years observed from date of first visit – end date  OR  >2 years of 2-6monthly visits |
| --- | --- |
| Visit B | Rheumatologist, immunologist or nephrologist in MBS or NAP:  **4** or more visits in 12-month period  OR  Total regular visits (defined as 6-24monthly) equalling number of years in data  OR  >2 years of 2-6monthly visits |
| Visit C | >=3 visits ever to rheumatologist, immunologist, nephrologist or general physician |
| Visit D | >=2 visits to rheumatologist, immunologist, nephrologist or general physician |

### **Supplementary Material S4:** Overall algorithm

| Dataset(s) | Certain SLE rule | Uncertain SLE rule |
| --- | --- | --- |
| Hospital | ≥ 2 admissions with SLE AND more admissions with SLE than any non-SLE SARD.  OR  One admission with SLE AND existing non-hospital SLE data point (MBS, NAP, PBS or Death items) AND more admissions with SLE than any non-SLE SARD. | ≥ 1 admission with SLE AND more admissions with SLE than any non-SLE SARD. |
| Death | SLE as the underlying cause of death, or as an additional cause, if a non-SLE SARD was not listed as underlying cause  AND  Non-death SLE data point (Hospital, MBS, NAP or PBS items) OR died prior to 2014. | SLE as the underlying cause of death, or as an additional cause, if a non-SLE SARD was not listed as underlying cause. |
| MBS and NAP: visits | ≥ 3 visits to a rheumatologist, immunologist or nephrologist in one year  OR  Regular (6-24 monthly) visits to a rheumatologist, immunologist or nephrologist totalling the same number as years observed in the data  OR  ≥ 2 years of visits to a rheumatologist, immunologist or nephrologist at 2-6 monthly intervals. | ≥ 3 visits to a rheumatologist, immunologist, nephrologist or general physician. |
| PBS | MPR of > 0.8 for > 20, 15 or 12 months (hydroxychloroquine, azathioprine, mycophenolate, respectively. | ≥ 2 dispensing of hydroxychloroquine, mycophenolate or azathioprine  MPR calculation, daily doses and PBS codes: Supplementary Material S4. |
| MBS: pathology | ≥4 dsDNA and complement tests. | ≥3 dsDNA and complement tests. |

### **Supplementary Material S5:** Selected sensitivity analyses of entire algorithm

**Hospital, death and certain SLE outpatient criteria sensitivity analyses**

| **Criteria being tested** | **Changed criteria** | **Remaining criteria** | **Interpretation** |
| --- | --- | --- | --- |
| **Hospital** | Hospital A | Death A, Med A, Visit A, Path A | Approx 13% difference. Review of sample of 5% cases did not clearly demonstrate any differences in terms of demographics or health utilisation. Given international SLE algorithms suggest Hosp A (>1 SLE coded event needed) is more specific, Hosp A used for certain, Hosp B for uncertain criteria. |
|  | Hospital B |  |  |
|  | Hospital C | Death A, Med A, Visit A, Path A | Within 5% of hospital A. Therefore, hospital A chosen for simplicity. |
| **Death** | Death A | Hosp A, Med A, Visit A, Path A | < 2% difference. Individual cases not reviewed. Death A used for certain, Death B uncertain. |
|  | Death B |  |  |
| **Visits** | Visit A | Death A, Med A, Hosp A, Path A | < 2% difference. Individual cases not reviewed. More inclusive (Visit A) criteria used. |
|  | Visit B |  |  |
| **Meds** | Med A | Death A, Visit A, Hosp A, Path A | 2.2% difference. Individual cases not reviewed. More inclusive (Med A – lower AZA and MMF doses) criteria used, to include lower-weight individuals or others on lower dose AZA/MMF.i |
|  | Med B |  |  |
| **Path** | Path A | Death A, Visit A, Hosp A, Med A | Approx 12% difference. Review of sample of 5% cases did not clearly demonstrate any differences in terms of demographics or health utilisation. After consultation with clinical expert group, Path A used, as frequency of pathology testing likely matches visits, so four was chosen as this represents the minimum visit count to meet certain SLE, and three represents minimum visit for uncertain SLE. |
|  | Path B |  |  |

**Uncertain SLE outpatient criteria sensitivity analyses**

| **Criteria being tested** | **Changed criteria** | **Remaining criteria** | **Interpretation** |
| --- | --- | --- | --- |
| **Visits** | Visit C | Hosp B, Death B,  Path B, Med C | <1% difference. Individual cases not reviewed. More inclusive criteria used. |
|  | Visit D |  |  |
| **Meds** | Med C | Hosp B, Death B,  Path B, Visit D | Approx 3.7% difference. Individual cases not reviewed. More inclusive criteria used. |
|  | Med D |  |  |
| **Path** | Path B | Hosp B, Death B,  Med C, Visit D | Approx 30% difference. On review of individual cases, Path C (2 dsDNA tests) had a higher proportion of individuals who had never met hospital criteria or only just met medication criteria. This was considered to represent a different group, perhaps with an alternate SARD. Path B used. |
|  | Path C |  |  |

### **Supplementary Material S6:** Death criteria detail

The same ICD10-AM codes listed for hospital criteria were used in selecting death records.

The below two criteria were tested in the overall sensitivity analyses.

| Death A | SLE as underlying cause of death OR SLE in contributing causes of death if no other ARD also listed in causes  + at least one other non-death data point, or date of death pre-01.01.2014 |
| --- | --- |
| Death B | SLE as underlying cause of death OR SLE in contributing causes of death if no other ARD also listed in causes |

### **Supplementary Material S7:** Medication criteria detail

The following codes were used to identify medications to include or exclude SLE

| **PBS: item codes for SLE case identification** | |
| --- | --- |
| Hydroxy- chloroquine | "01512N" "12003R" |
| Azathioprine | "02687K" "02688L" |
| Mycophenolate | "01836P" "01837Q" "01839T" "02150E" "02193K" "06208R" "06209T" "06364Y" "06369F" "06370G" "08649F" "08650G" "08651H" "08652J" "08653K" "09500B" "09501C" "09502D" "09503E" "09504F" "13856G" "13884R" "13938N" "14000W" "14037T" "14071N" |
| **PBS: item codes for exclusion of SLE if >=3 (per drug) present** | |
| Upadacitinib | "11979L" "11989B" "12621G" "12624K" "12625L" "12648Q" "12685P" "12827D" "12828E" "12829F" "12831H" "12835M" "12836N" "13249H" "13250J" "13256Q" "13262B" "13265E" "13343G" "13350P" "13746L" "13762H" "13768P" "13771T" "14125K" |
| Tofacitinib | "10511F" "10517M" "11675L" "11690G" "12556W" "12557X" "12588M" "12589N" "12720L" "13345J" "13349N" "13730P" "13737B" "13738C" "13755Y" "13757C" "13770R" "13776C" |
| Baricitinib | "11437Y" "11442F" "11443G" "11447L" "12708W" "12730B" "13689L" "13702E" |
| Ustekinumab | "09304Q" "09305R" "10767Q" "10774C" "11164N" "11178H" "11182M" "12662K" "12664M" "12669T" "13255P" "13261Y" "13272M"  [14] "13273N" "13781H" "13789R" "13804M" "13805N" |
| Secukinumab | "10425Q" "10494H" "10890E" "10893H" "10894J" "10895K" "10898N" "10899P" "10900Q" "10901R" "10906B" "10910F" "12297F" "12307R" "12321L" "14146M" "14154Y" "14161H" "14164L" |
| Ixekizumab | "11032P" "11033Q" "11623R" "12209N" "12217B" |
| Guselkumab | "11614G" "12568L" "12590P" |
| Tocilizumab | "01419Q" "01423X" "01464C" "01476Q" "01481Y" "01482B" "09657G" "09658H" "09659J" "09671B" "09672C" "09673D" "10056G" "10058J" "10060L" "10064Q" "10068X" "10071C" "10072D" "10073E" "10077J" "10078K" "10079L" "10081N" "10951J" "10954M" "11565Q" "11567T" "11720W" "11721X" "11722Y" "11725D" "11730J" "11734N" "11741Y" "11742B" "11743C" "11744D" "11748H"  "11750K" "12083Y" "12084B" "12085C" "12086D" "12090H" "12094M" "12095N" "12099T" "12102Y" "12105D" "12692B" "12693C""12694D" "12705Q" "12706R" "12707T" "12713D" "12714E" "12726T" "12727W" "12728X" "12747X" "12761P" "12762Q" "12763R"  "12766X" "12767Y" "12768B" "12775J" "12787B" "12791F" "12792G" "12794J" "12795K" "12796L" "12802T" "12805Y" "12806B""12810F" "12811G" "13299Y" "13301C" "13304F" "13305G" "13306H" "13311N" "13312P" "13315T" "13323F" "13324G" "13329M" "13330N" "13338B" "13339C" "13684F" "13685G" "13690M" "13696W" "13715W" "13716X" "13720D" "13731Q" |
| Anakinra | "08773R" "08774T" "10263E" "10264F" |
| Abatacept | 01220F" "01221G" "05605B" "09621J" "11068M" "11092T" "11684Y" "11693K" "12680J" "12738K" "12756J" "12758L" "13034B" "13037E" "13705H" "13725J" "13726K" "13727L" |
| Golimumab | "03426H" "03427J" "03428K" "03429L" "03430M" "03431N" "03432P" "03433Q" "03434R" "03435T" "03436W" "03437X" "11361Y" "11365E" "11372M" "11373N" "11375Q" "11376R" "11381B" "11382C" "11502J" "11516D" "11521J" "11538G" "11560K" "12703N" "12744R" "13699B" "13706J |
| Certolizumab | "03425G" "10137M" "10238W" "10892G" "10896L" "10897M" "10904X" "10905Y" "10909E" "11318Q" "11319R" "11320T" "11321W" "11322X" "11323Y" "11324B" "11325C" "11326D" "12005W" "12013G" "12027B" "12028C" "12040Q" "12063X" "12691Y" "12702M" "12712C" "12724Q" "12742P" "12743Q" "13701D" "13735X" |
| Etanercept | "01954W" "01963H" "01964J" "03445H" "03446J" "03447K" "03448L" "03449M" "03450N" "05733R" "05734T" "05735W" "06367D" "08637N" "08638P" "08778B" "08779C" "08861J" "08862K" "09035M" "09036N" "09037P" "09038Q" "09081Y" "09082B" "09083C" "09084D" "09085E" "09086F" "09087G" "09088H" "09089J" "09090K" "09091L" "09429G" "09430H" "09431J" "09455P" "09456Q" "09457R" "09458T" "09459W" "09460X" "09461Y" "09462B" "09615C" "09641K" "11196G" "11197H" "11198J" "11201M" "11202N" "11204Q" "11207W" "11208X" "11211C" "11215G" "11216H" "11217J" "11218K" "11219L" "11220M" "11221N" "11222P" "11223Q" "11224R" "11225T" "12675D" "12676E" "12679H" "12716G" "12718J" "12734F" "12735G" "12736H" "12737J" "12740M" "12750C" "12757K" "12838Q" "12845C" "12848F" "12852K" "12853L" "12862Y" "12863B" "12870J" "12871K" "12873M" "12874N" "12875P" "12880X" "12881Y" "12882B" "12893N" "12894P" "12906G" "12907H" "12908J" "12909K" "13294Q" "13295R" "13308K" "13319B" "13326J" "13332Q" "13687J" "13693Q" "13697X" "13698Y" "13707K" "13708L" "13733T" "13751R" "13774Y" |
| Adalimumab | "05281Y" "05282B" "05283C" "05284D" "08737W" "08741C" "08961P" "08962Q" "08963R" "08964T" "08965W" "08966X" "09033K" "09034L" "09077R" "09078T" "09099X" "09100Y" "09101B" "09102C" "09103D" "09104E" "09186L" "09187M" "09188N" "09189P" "09190Q" "09191R" "09425C" "09426D" "09427E" "09428F" "09661L" "09662M" "09663N" "09678J" "09679K" "09680L" "10389T" "10396E" "10397F" "10399H" "10400J" "10404N" "10412B" "10413C" "10419J" "10420K" "10422M" "10944B" "10945C" "10955N" "10960W" "10961X" "10972L" "11121H" "11127P" "11132X" "11133Y" "11137E" "12325Q" "12326R" "12327T" "12328W" "12329X" "12330Y" "12331B" "12332C" "12333D" "12334E" "12335F" "12336G" "12337H" "12338J" "12339K" "12340L" "12341M" "12342N" "12343P" "12344Q" "12345R" "12346T" "12347W" "12348X" "12349Y" "12350B" "12351C" "12352D" "12353E" "12354F" "12355G" "12356H" "12357J" "12358K" "12359L" "12360M" "12361N" "12362P" "12363Q" "12364R" "12365T" "12366W" "12367X" "12368Y" "12369B" "12370C" "12371D" "12372E" "12373F" "12374G" "12375H" "12376J" "12377K" "12378L" "12379M" "12380N" "12381P" "12382Q" "12383R" "12384T" "12385W" "12386X" "12387Y" "12388B" "12389C" "12390D" "12391E" "12392F" "12393G" "12394H" "12395J" "12396K" "12397L" "12398M" "12399N" "12400P" "12401Q" "12402R" "12403T" "12404W" "12405X" "12406Y" "12407B" "12408C" "12409D" "12410E" "12411F" "12412G" "12413H" "12414J" "12415K" "12416L" "12417M" "12418N" "12419P" "12420Q" "12421R" "12422T" "12423W" "12424X" "12425Y" "12426B" "12427C" "12428D" "12429E" "12430F" "12431G" "12432H" "12433J" "12434K" "12435L" "12436M" "12437N" "12438P" "12439Q" "12440R" "12441T" "12442W" "12443X" "12444Y" "12445B" "12446C" "12447D" "12448E" "12449F" "12450G" "12451H" "12452J" "12453K" "12454L" "12455M" "12524E" "12674C" "12678G" "12686Q" "12690X" "12695E" "12696F" "12697G" "12698H" "12699J" "12700K" "12701L" "12715F" "12719K" "12729Y" "12731C" "12732D" "12733E" "12741N" "12749B" "12752E" "13208E" "13209F" "13210G" "13211H" "13212J" "13213K" "13214L" "13215M" "13216N" "13217P" "13218Q" "13219R" "13220T" "13221W" "13222X" "13223Y" "13224B" "13225C" "13226D" "13227E" "13228F" "13229G" "13230H" "13292N" "13293P" "13686H" "13691N" "13692P" "13703F" "13704G" "13721E" "13722F" "13732R" "13744J" "13754X" "13763J" "13764K" "14221L" "14222M" "14223N" "14234E" "14235F" "14242N" "14243P" "14244Q" "14245R" "14251C" "14252D" "14253E" "14261N" "14262P" "14263Q" "14272E" "14273F" "14274G" "14275H" "14276J" "14283R" "14284T" "14285W" "14286X" "14294H" "14295J" "14586Q" "14587R" "14591Y" "14622N" "14628X" "14629Y" |
| Infliximab | "04284L" "05753T" "05754W" "05755X" "05756Y" "05757B" "05758C" "06397Q" "06398R" "06448J" "06496X" "09612X" "09613Y" "09617E" "09654D" "09674E" "10057H" "10067W" "10184B" "10196P" "11389K" "11396T" "11399Y" "11400B" "11412P" "11423F" "11424G" "11432Q" "11445J" "11448M" "11449N" "11450P" "11459D" "11461F" "11481G" "11482H" "11483J" "11486M" "11487N" "11488P" "11489Q" "11490R" "11497D" "11498E" "11514B" "11515C" "11590B" "11595G" "11605T" "11606W" "11796W" "11797X" "12550M" "12551N" "12552P" "12553Q" "12554R" "12555T" "12560C" "12561D" "12566J" "12567K" "12575W" "12576X" "12577Y" "12584H" "12585J" "12586K" "12587L" "12597B" "12677F" "12687R" "12688T" "12689W" "12717H" "12748Y" "12751D" "12753F" "13047Q" "13048R" "13049T" "13050W" "13054C" "13055D" "13056E" "13057F" "13058G" "13060J" "13061K" "13062L" "13065P" "13066Q" "13067R" "13069W" "13070X" "13072B" "13073C" "13074D" "13075E" "13076F" "13077G" "13078H" "13700C" "13723G" "13724H" "13734W" "13765L" "13778E" |

The medication/medicines possession ratio (both names used interchangeably in the literature) was calculated using standard pharmacoepidemiological methods, which are outlined in detail elsewhere [1, 2]. To summarise, our approach was as follows:

1. Daily dose chosen. The WHO defines the Defined Daily Dose (DDD) as the assumed average maintenance dose per day for a drug used for its main indication in adults. The WHO sets DDD for most drugs in the Anatomical Therapeutic Chemical classification scheme.
   a. The WHO DDD for azathioprine is 150mg daily
   b. The WHO DDD for mycophenolate mofetil is 2000mg daily
   c. The WHO DDD for hydroxychloroquine is 516mg daily (this is based on treatment for malaria)

The WHO DDD were refined into more clinically relevant daily doses of:

| Lower bound daily dose hydroxychloroquine | 200mg |
| --- | --- |
| Upper bound daily dose hydroxychloroquine | 400mg |
| Lower bound daily dose azathioprine | 50mg |
| Upper bound daily dose azathioprine | 150mg |
| Lower bound daily dose mycophenolate | 1000mg (mycophenolate mofetil) or 720mg (mycophenolate sodium) |
| Upper bound daily dose mycophenolate | 2000mg (mycophenolate sodium) or 1440mg (mycophenolate sodium) |

2. The MPR was calculated for each medication and daily dose as follows:

- Covered Days: For each medication dispensing, the number of days covered was determined by:

$$Covered Days= \frac{Quantity Supplied \times Unit Strength}{Daily Dose}$$

- Total Days: Defined as the interval from the date of the first dispensing to the date of the last dispensing
- Total Covered Days: defined as the sum of Covered Days from each dispensing within the observation period (note the date of the last dispensing is the end date, therefore the medication dispensed in the last dispending are not included). This accounts for stockpiling, as if the interval between two dispensing events was shorter than the covered days from the preceding dispensing, the surplus days were carried over to the subsequent period as they were included in Total Covered Days.
- The MPR was computed as:

$$MPR= \frac{Total Covered Days}{Total Days}$$

This calculation was performed sequentially across dispensing events to identify the longest continuous period during which an individual's MPR remained >0.8. Specifically, MPRs were calculated for all dispensing sequences (e.g., scripts 1-2, 1-3, 1-4, etc.), and then repeated for all possible dispensing combinations (e.g., scripts 2-3, 2-4, 3-4, 3-5 etc.). The longest period for which an individual maintained an MPR >= 0.8, for each scenario was recorded. This accounts for the fact individuals may have periods of greater and lesser medication adherence, particularly given the long median observation period of 11 years.

**Approach to Hydroxychloroquione Daily Dosing**

The recommended dosing of hydroxychloroquine for SLE is 5mg/kg, and tablets are in 200mg only. Weekly dosing differs by patient and can include not only 200mg or 400mg daily doses but also alternating daily doses and other creative measures to reach approximately 5mg/kg/day across a week. It is not possible to know from the NHDH the actual prescribed dose for everyone, we have therefore chosen 200mg and 400mg as common doses to test. This is still complex, as the problem with using a DDD of 200mg for all participants is that those who had an actual prescribed dose of 400mg would more easily meet the 200mg MPR >0.8 for 20 months even if they had a less persistent usage, due to the way the medication scripts would be counted forward as oversupply. Therefore, to circumvent this, all individuals with a script count >4 annually (5 HCQ scripts per year being the lower range to meet MPR >0.8 for 400mg dosage) had to meet the higher dose cutoff, whereas others could meet a dose of 200mg/day. We acknowledge some individuals take less than 200mg/day using alternate daily dosing, and these cases will be captured by the ‘uncertain’ criteria.

The following criteria were tested in the overall sensitivity analyses.

| Med A | HCQ: >20 months of MPR >0.8. DDD = 200mg unless >7 scripts in a year then DDD = 400mg OR  MMF: >12 months of MPR >0.8. DDD=1000mg/720mg OR  AZA: >15 months of MPR >0.8. DDD = 50mg |
| --- | --- |
| Med B | HCQ: >20 months of MPR >0.8. DDD = 200mg unless >7 scripts in a year then DDD = 400mg OR  MMF: >12 months of MPR >0.8. DDD= 2000mg/1440mg OR  AZA: >15 months of MPR >0.8. DDD = 150mg |
| Med C | >2 scripts of HCQ, MMF or AZA dispensed, |
| Med D | > 3 scripts of HCQ, MMF or AZA dispensed. |

### **Supplementary Material S8:** Pathology criteria detail

The following MBS codes were used to identify SLE-related MBS claims. Given that dsDNA and complement levels are often tested in tandem, we defined a combined dsDNA and complement test as a code for each occurring in the same month (exact dates are not in the NHDH unless by exception).

| **MBS: original item code** | |
| --- | --- |
| 71099 | Double-stranded DNA antibodies - quantitation by 1 or more methods other than the Crithidia method |
| 71083, 71085 | Quantitation of complement components C3 and C4 or properdin factor B - 1 test;  2 tests described in item 71083 |

The below three criteria were tested in the overall sensitivity analyses.

| Path A | dsDNA + C3/C4 in same month x 4 |
| --- | --- |
| Path B | dsDNA + C3/C4 in same month x 3 |
| Path C | dsDNA + C3/C4 in same month x 2 |

### **Supplementary Material S9:** Demographic and disease characteristics

**Demographic characteristics**

| **Variable** | **Data source** | **Explanation** |
| --- | --- | --- |
| Gender | NHDH universal demographic variables | Supplied by AIHW, extracted from combination of different linked datasets, to generate single value per person. |
| Remoteness area | NHDH universal demographic variables | Supplied by AIHW, extracted from combination of different linked datasets, to generate single value per person. Remoteness area as defined by Australian Bureau of Statistics, divide Australia into five classes of remoteness which are characterised by a measure of relative geographic access to services (https://www.abs.gov.au/statistics/standards/australian-statistical-geography-standard-asgs-edition-3/jul2021-jun2026/remoteness-structure/remoteness-areas). |
| IRSD quintile | NHDH universal demographic variables | Supplied by AIHW, extracted from combination of different linked datasets, to generate single value per person. Index of relative socioeconomic disadvantage, as defined by ABS, summarises a range of information about the economic and social conditions of people and households within an area. It includes only indicators of relative disadvantage. Areas are ranked according to their IRSD score and then split into five equal groups (quintiles), with quintile 1 containing the most disadvantaged areas and quintile 5 the least disadvantaged. |
| Region of birth | Hospital data | Hospital data includes country of birth, using the Standard Australian Classification of Countries (SACC). The NHDH only has the SACC to the Major Group level, of which there are 9. These have been further aggregated in our results, by combining North and South Europe, and North-East, South-East and Central Asia. This data is only available for individuals who have, firstly, had a hospital admission (any cause), and secondly provided this data. Data is missing for ~12-15% of the cohort. |

**Health utilisation and disease characteristics**

| **Variable** | **Data source** | **Explanation** |
| --- | --- | --- |
| Proportion receiving dialysis | Hospital and MBS | Admission with code Z49.0, Z49.1, Z49.2, Z99.2, T85.71  MBS item numbers: 13105, 13109, 13110, 13104, 13103, 13100, 13106 |
| Proportion receiving renal transplant | Hospital | Admission with code Z49.0, T86.1 |
| Proportion hospitalised for childbirth | Hospital | Admission with code Z37* |
| Proportion seeing GP | MBS | MBS item with BTOS 101, 102 |
| Proportion seeing Obstetrician / Gynaecologist | MBS | MBS visit to obstetrician (SPR REG SPEC 53, 218) and BTOS 200, 300 |
| Proportion seeing dermatologist | MBS | MBS visit to dermatologist (SPR REG SPEC 52, 401) and BTOS 200, 103 |
| Proportion seeing ophthalmologist | MBS | MBS visit to ophthalmologist (SPR REG SPEC 54, 406 and BTOS 200, 300 |
| Proportion receiving automated visual field testing | MBS | MBS item number 1121, 10940 |

### **Supplementary Material S10:** SLE annual incidence rates

The table below presents the annual incident cases and the annual incidence rate per 100,000 person years, for 2015- 2019

| **Table 5: Incident certain and overall SLE cases (incidence / 100,000 person years), per state** | | | | | | | | | | | |
| --- | --- | --- | --- | --- | --- | --- | --- | --- | --- | --- | --- |
|  | Certain SLE cases (incidence / 100,000 person-years) | | | | | | Overall SLE cases (incidence / 100,000 person-years) | | | | |
|  |  | 2015 | 2016 | 2017 | 2018 | 2019 | 2015 | 2016 | 2017 | 2018 | 2019 |
|  | ACT | 38 (9.6) | 44 (10.9) | 31 (7.5) | 41 (9.6) | 38 (8.7) | 69 (17.4) | 78 (19.3) | 50 (12) | 65 (15.3) | 64 (14.7) |
|  | NSW | 573 (7.5) | 569 (7.4) | 517 (6.6) | 546 (6.9) | 545 (6.8) | 887 (11.6) | 847 (11) | 848 (10.8) | 883 (11.1) | 937 (11.6) |
|  | QLD | 287 (6.0) | 259 (5.3) | 227 (4.6) | 245 (4.9) | 230 (4.5) | 445 (9.3) | 393 (8.1) | 380 (7.7) | 415 (8.3) | 394 (7.7) |
|  | SA | 122 (7.2) | 95 (5.5) | 104 (6) | 101 (5.8) | 88 (5) | 188 (11.1) | 146 (8.5) | 197 (11.4) | 187 (10.7) | 173 (9.8) |
|  | TAS | 26 (5) | 19 (3.7) | 13 (2.5) | 20 (3.7) | 17 (3.1) | 39 (7.6) | 37 (7.1) | 27 (5.1) | 38 (7.1) | 28 (5.1) |
|  | VIC | 355 (5.9) | 376 (6.1) | 407 (6.5) | 389 (6.1) | 464 (7.1) | 545 (9) | 586 (9.5) | 639 (10.1) | 646 (10.1) | 747 (11.4) |

*Limitation to consider in incidence calculation: in* common with other studies estimating incidence from healthcare utilisation data, we were unable to determine the true date of disease onset. Disease onset may precede the first hospital admission but follow earlier contacts, such as outpatient rheumatology visits, often resulting in a lag of several years between diagnosis and case capture. This limitation primarily affects estimates of annual incidence rather than period prevalence. However, in the absence of major policy or systemic changes that would significantly alter healthcare utilisation patterns, this lag is likely to be consistent across calendar years. This is supported by the relatively stable annual incidence rates observed from 2015 to 2019, suggesting that this limitation does not materially bias temporal trends.

**References**

1.	Andrade, S.E., et al., *Methods for evaluation of medication adherence and persistence using automated databases.* Pharmacoepidemiology and drug safety, 2006. **15**(8): p. 565-574.

2.	Rasmussen, L., et al., *Core concepts in pharmacoepidemiology: measures of drug utilization based on individual‐level drug dispensing data.* Pharmacoepidemiology and drug safety, 2022. **31**(10): p. 1015-1026.
